# Screening and Assessment for Psychological Distress among Burn Survivors

**DOI:** 10.3390/ebj3010008

**Published:** 2022-02-03

**Authors:** Valerie G. Loehr, William F. Goette, Kimberly Roaten

**Affiliations:** Department of Psychiatry, UT Southwestern Medical Center, Dallas, TX 75390-8898, USA; william.goette@utsouthwestern.edu (W.F.G.); kimberly.roaten@utsouthwestern.edu (K.R.)

**Keywords:** assessment, mental health screening, psychological distress, symptom screening, symptom severity, burn injury

## Abstract

Given the high rates of psychological distress after burn injury, thorough screening and assessment for psychosocial factors and psychiatric pathology should be routinely completed for individuals with burn injuries. Burn survivors experience unique psychosocial changes and injury sequelae, such as body image concerns, trauma-related pathology, and itching. Screening for these factors is integral to understanding how these may be contributing to psychological distress. Proactively identifying distress and psychiatric pathology is important to optimize physical and emotional outcomes. The aim of this manuscript is to summarize information about the available screening and assessment tools for psychological distress among burn survivors.

## 1. Introduction

Between 2009 and 2018, there were 221,519 individuals who were admitted to hospitals in the US for burn injuries. Children up to age 16 comprised 22.5% of total burns, while adults ages 20–59.9 made up 56% of burns [1]. Burn trauma can affect and worsen the physical and mental health of survivors and present significant social challenges, especially for those who experience larger burns.

### 1.1. Pre- and Post-Burn Psychopathology

While the extent and nature of the injury will affect adjustment and quality of life, pre-injury functioning and psychopathology are important factors in predicting burn outcomes, including length of stay in the hospital, complications, overall increased morbidity, and development of acute mental disorders during the hospitalization [2,3,4,5]. Preexisting psychiatric disorders and symptoms are common in burn patients [6], with an increased prevalence of pre-burn depression and substance use compared to the general population [7]. Burn survivors without a preinjury mental health diagnosis showed a greater number of subthreshold mental health symptoms prior to their injuries than a normative sample [6]. Increased rates of subthreshold mental health symptoms may be related to greater difficulties in taking care of their safety and well-being, excessive risk-taking, or being in less safe environments. Burn survivors, with or without a premorbid psychiatric diagnosis, are a vulnerable population. Identifying pre-morbid psychopathology upon admission is important to ensuring a comprehensive approach to patient care to improve burn outcomes.

While preexisting mental health symptoms can contribute to sustaining a burn injury, post-injury psychiatric symptoms are also common. Nearly one half of burn survivors develop a mental health disorder after their injury [8], including anxiety, PTSD, depression, and delirium. Children with burn injuries have significantly greater rates of post-burn mental health admissions, including mood and anxiety disorders [9]. Preexisting illness and post-injury mental health disorders may make it more difficult to effectively adapt to life after the injury and increase risk for suicidality [10].

#### 1.1.1. Anxiety and Trauma

Anxiety is a common response in burn recovery and to the treatments necessary to heal burned tissue. Anxiety is often experienced in conjunction with the physical trauma and sequalae of the burn, such as with pain and itching. The origin of anxiety may also be related to the trauma itself; therefore, it is important to understand the nature and source of the symptoms and tailor treatment accordingly. Depending on the circumstances related to the injury, burn survivors are at risk of developing acute stress syndrome and post-traumatic stress disorder (PTSD), with symptoms including intrusive memories, nightmares, avoiding thoughts and reminders of the event, numbing or dissociating, hyperarousal, and irritability. As per the Diagnostic and Statistical Manual of Mental Disorders, Fifth Edition (DSM-5), 10% of severe burn survivors meet criteria for acute stress disorder. Nearly half of the individuals diagnosed with PTSD initially present with acute stress disorder [11], indicating the need to assess trauma-related psychopathology soon after the injury and post-hospitalization.

#### 1.1.2. Depression

Traumatically injured and medically ill patients have higher rates of depression than the general population [12]. Burn survivors, in particular, may be at increased risk, due to impaired or loss of functioning, changes in physical appearance, difficulty managing pain, or time away from loved ones due to extended hospitalization and physical rehabilitation. Studies have found a depression rate of 30–35% among burn patients during their hospitalization using validated questionnaires, and some studies report higher prevalence, particularly following discharge [13]. 

#### 1.1.3. Alcohol and Substance Use Disorders

A significant number of burn injuries occur in the context of acute alcohol or drug intoxication. Studies found that 40–55.5% of all burn injuries had a positive screen for at least one drug of abuse [14], and nearly two thirds with a self-inflicted injury had a drug screening positive for illicit drugs [15]. There is also concern that ingesting alcohol just prior to the injury may result in physiological changes that negatively affect the body’s ability to heal [16], and illicit drug use prior to the accident is associated with an extended ICU stay [15]. 

#### 1.1.4. Suicide

Suicide by fire, known as self-immolation, accounts for 1% of all suicides in high-income countries (United States and Western Europe) [17]. Self-immolation patients have a higher percentage total burn surface area (TBSA), higher mortality [18], and greater rate of a premorbid psychiatric or substance use disorder [19]. These patients are at a greater risk of subsequent suicide attempts and death [20,21], and many of these patients do not receive psychiatric follow-up [22].

#### 1.1.5. Delirium

Delirium is common in acute burn treatment and causes disturbances in attention, consciousness, and cognition, and can cause psychomotor agitation or retardation, impairments in sleep–wake cycle, emotional disturbances, and hallucinations or delusions. Consequently, the symptoms of delirium can be difficult to distinguish from depression, psychosis, or dementia. Estimates of delirium following burn injury vary from 13–39% among general or older burn patients [23,24,25,26,27] to as much as 77% among ventilated burn patients [26]. Factors that increase the risk of delirium among burn patients include older age, number of operations/wound care procedures completed under anesthesia, provision of intensive care, physical impairment, use of anticholinergic drugs, exposure to benzodiazepines, and increased American Society for Anesthesiologists scores [26,28,29]. Due to the nature of delirium and burn injury, the burn patient’s neuropsychiatric symptoms may be transitory, wax and wane, or persist for weeks. Delirium is an organic process and is a result of a disease process, medication side effects, drug withdrawals, or other factors, including pain or malnutrition. Consequently, it is important to know the patient’s baseline mental functioning and to identify and treat the underlying causes of delirium. 

### 1.2. Psychosocial Factors Unique to Burn: Pain Control, Pruritus, Body Image, and Integration into the Community 

Burns that require hospitalization can result in numerous physical and psychosocial challenges. Burn survivors face unique short- and long-term consequences, such as pain, body image changes, and itching, which can significantly impact quality of life. Pain control for burn survivors in the acute care setting is challenging, given the nature of tissue damage and the procedures required for wound care and therapies. Adequate pain control results in improved wound healing, improved sleep, improved quality of life, and participation in therapies and activities [27], while inadequately treated pain can depress the cardiovascular system and lead to a stress response, which can create biochemical and metabolic disturbances [28]. Pruritus, or itching, is common during the healing phase of burn injury and can last for months or years. Itching can be so severe that it disrupts sleep [29], the activities of daily living [30], and psychosocial well-being [31]. These factors can further delay healing and intensify the itching sensation, and scratching can interrupt the healing process if it causes an infection in the area.

A major burn injury can result in considerable damage to skin integrity and, consequently, lead to hypertrophic scarring. Deep burns can lead to impaired functioning that can result in loss of functionally and cosmetically important body parts [32]. Disfigurement of socially visible areas (e.g., face) and ‘hidden’ areas (e.g., genitalia) and body image dissatisfaction have been shown to be related to the development and maintenance of psychological distress and lower self-esteem after burn injury [33].

These factors may result in challenges in integrating back into the community. Community integration is the ability to fully integrate into their roles at home and the community and the ability to participate in leisure and productive activities at home, work, and school [34]. Long-term psychosocial well-being, satisfaction, and adaptation depends on how well they can successfully integrate back into their community. 

### 1.3. Aim

Identifying premorbid and current psychiatric symptoms and psychosocial factors that can significantly affect quality of life and adaptive coping is important for optimizing recovery. Multiple studies have shown that receiving psychological support in burn units decreases length of stay and morbidity [35]. The aim of this manuscript is to summarize information about the available screening and assessment tools for psychological distress and psychosocial factors related to burns, which providers and staff can employ in their practice.

## 2. Assessment Measures

### 2.1. Outcomes

Table 1 and Table 2 provide information about each measure, including the number of questions, age range, language(s), and internal consistency. Table 1 provides utility statistics, based on the reported psychometrics reviewed for each scale when possible. These include the reliable change index (RCI), the recommended cut-off score for screening the condition of interest, the associated likelihood ratio of a positive screening (LR+), and positive predictive probability (PPV) of the condition of interest, given a positive screening result. The RCI is useful for repeat assessments and measuring response to treatment, because it generates a sufficient change in scores to be considered a reliable change (i.e., one large enough to not be due to measurement error alone). The PPV corresponds to the post-test probability of an individual having the condition of interest, given a score on the screening measure that exceeds the cut-off score. The PPV considers the prevalence of the condition of interest, and it should be noted that the single cut-off score should only be used for gross screening, as scores even further above the cut-off likely also have increased PPV. Scales are reviewed next, with an emphasis on their psychometric properties, including reliability and validity in burn populations where possible. None of the scales listed in Table 1 have been validated for burn survivors, though they are frequently used with this population.

### 2.2. Depression Scales

A recent systematic literature review on the assessment of depression in burn populations recommended formal, routine depression screenings, but highlighted the relative dearth of psychometric data on the numerous depression inventories [36]. Multiple factors affect the measurement of risk and symptom severity, including the depression scale used [37], metric for burn severity [38], and pre-morbid psychiatric history [39].

#### 2.2.1. Beck Depression Inventory-II

The Beck Depression Inventory-II (BDI-II) is a 21-item, self-report depression severity rating scale available through Pearson (starter kits begin at 98.40 USD). Administration and scoring time varies, but generally requires between 5 and 10 min. The BDI-II is commonly used both clinically and in research, though much less research is available specific to the BDI-II in burn populations.

A meta-analysis summarizing 99 studies, involving a total of 31,413 participants, found that the BDI-II has an internal consistency of 0.89 but that this varied depending on whether the sample was clinical (α = 0.91) or non-clinical (α = 0.88) [40], which is similar to the meta-analytic estimate (α = 0.91) found by other researchers [41]. In the subset of studies that reported the test-retest reliability of the BDI-II, the average test-retest reliability coefficient was 0.75, with a median retest interval of 6 weeks [40]. In a post-burn study [13], BDI-II reliabilities were all greater than 0.90 at one month, one year, and two years after discharge, which is consistent with these meta-analytic results from general populations. Meta-analytic factor analysis of the BDI-II supports two dominant interpretations of the scale: either a single global depression factor (α = 0.91), or a cognitive (α = 0.70) and somatic (α = 0.77) oblique factor model (*r* = 0.81) [41]. The BDI-II scores routinely demonstrate strong positive correlations with other depressive measures: *r* = 0.72 with the Center for Epidemiological Studies depression scale, *r* = 0.76 with the Zung depression rating scale, and *r* = 0.74 with the Hamilton depression scale [40].

The meta-analytic diagnostic accuracy of the BDI-II generally supports the use of a cutoff score between 13 and 15 [40,42]. The meta-analytic diagnostic accuracy of the BDI-II supports a cutoff of 14.5 (sensitivity = 0.86, specificity = 0.78) [42], which is similar to the 13 cutoff score reported by [40], which produced a sensitivity of 0.83 and specificity of 0.76. 

As reported in Table 1, a change of around 3 to 4 points on the BDI-II is needed to be considered a reliable change over time. Additionally, having a raw score above 13 increases the probability of having depression from 0.07 (i.e., the midpoint prevalence estimate from [37]) to 0.22, which is an appreciable increase is probability. As the BDI-II can have raw scores as high as 63, scores that are well above the 13 cutoff threshold likely reflect an appreciably greater PPV. 

#### 2.2.2. Hamilton Rating Scale for Depression

This scale was originally proposed as a 21-item scale, whose first 17 items should be used in assessing depression as the latter four items were either unrelated to depression criteria, uncommon, or unrelated to depressive severity [43,44]. Since this original description, multiple variations of the Hamilton rating scale for depression (HAM-D) have been described, including the various 6-, 8-, 17-, 21-, 24-, 25-, 27-, and 36-item versions [44]. Both structured and unstructured HAM-D scales exist, though the recommendation is that structured forms provide better results [44]. Administration and scoring generally take approximately 5 min.

In a meta-analysis of the reliability of HAM-D [45], a favorable internal consistency of α = 0.79, inter-rater reliability of κ = 0.81 (ICC = 0.94), and test-retest reliability ranging from 0.65 to 0.98 were reported. In contrast, an earlier systematic review challenged the historical gold-standard status of the HAM-D and concluded that, while basic psychometric properties are consistently met, the items of the HAM-D are poorly designed and produce an undesirably multidimensional scale [46]. On the whole, the HAM-D has demonstrated repeated evidence of construct validity, as it is the most commonly used gold-standard test, against which other depression symptom inventories are compared [44,46].

Much of the research on the HAM-D’s sensitivity to depressive symptomatology is in the context of pharmaceutical research, rather than symptom progress tracking or population-level depression screening. As such, cut-off scores typically focus on quantifying severe depression or classifying the severity of depression (from mild to severe). Choice of which HAM-D scale to utilize is subject to clinical needs; however, the HAM-D_17_ is likely the most researched form, as it is consistent with the original 17 scored items. For the HAM-D_17_, scores between 8 and 16 reflect mild depression, 17 and 23 moderate depression, and scores of 24 or more severe depression [47]. Consistent with these cut-off classifications for depression severity, scores that change to become less than eight on the HAM-D_17_ should be considered as evidence of depression remission [48].

Among burn populations, relatively few studies have examined the HAM-D. One older study reported the utility of the HAM-D in screening for major depression in mothers of children with severe burns [49]. Notably, the study found that none of the children with burn injuries had major depression (6.3% had post-traumatic stress disorder, though) while, in contrast, 18.8% of mothers had major depression (with 12.5% having post-traumatic stress disorder) [49]. Another study examined the relationship between opioid analgesics and depressive symptoms on the HAM-D among burn patients [50]: controlling for age and total burn surface area, total opioid dose and HAM-D scores were positively correlated at a two-week follow-up (*r* = 0.33), and average total opioids dose was significantly greater in individuals with high HAM-D scores (raw score > 10).

#### 2.2.3. Center for Epidemiological Studies Depression Scale

This free-to-use, self-report depression scale was developed at the Center for Epidemiologic Studies at the National Institute of Mental Health [51] and has been used in multiple large-scale studies. The scale has also been studied in multiple international projects and among varying age groups. Administration and scoring generally take between 5 and 10 min.

The Center for Epidemiologic Studies Depression Scale (CES-D) has generally been shown to demonstrate the four-factor structure originally intended for capturing depressed affect (7 items), positive affect (4 items), somatic complaints (7 items), and interpersonal difficulties (2 items) among adolescents [52] and older adults [53]. Scores on the CES-D may be artificially elevated among women, due to differential item functioning [54]. Reliability is generally good, with internal consistency estimates around α = 0.85 being common [51,55], including among burn survivors: α = 0.92 [56].

A common cut-off score for the CES-D is 16 or higher. A recent meta-analytic diagnostic accuracy study of the CES-D found that, using this cut-off, the scale has a sensitivity of 0.87 and specificity of 0.70 for depression [57]. The authors of this meta-analysis, however, note that a better trade-off of sensitivity and specificity is obtained with a cut-off score of 20 (sensitivity = 0.83, specificity = 0.78) [57]. Based on these results, a positive score using a cut-off of 16 on the CES-D raises the probability of depression from 0.07 to 0.18, while a score above 20 raises the probability to 0.22 (see Table 1). 

With regard to burn populations, 27% of survivors produced a CES-D scores of 16 or greater [56]. They further report that individuals with active burn-related pain reported significantly greater CES-D scores (M_Pain_ = 17.02, SD_Pain_ = 13.48 versus M_NoPain_ = 10.93, SD_NoPain_ = 10.89) [56]. Utilizing a Korean version of the CES-D, depression scores were correlated with the patient scar assessment and brief burn-specific health scale [58]. Applying a cut-off of 25 or greater for classifying ‘definite depression’, about 50% of their sample had depression, and those with this high degree of depressive symptom endorsement also had higher average patient scar assessment scores [58].

#### 2.2.4. Patient Health Questionnaire

The patient health questionnaire (PHQ) was developed to be a self-report mental health screening instrument [59,60]; however, its nine depression items have emerged as a common depression screening inventory. The PHQ-9 and its short form, based on just two symptoms, the PHQ-2, are free to access. The PHQ-9 has been more extensively studied among burn survivors, though at least one study has examined the use of the PHQ-2. The PHQ can be accessed in various languages without cost at https://www.phqscreeners.com/. Administration time varies based on the version of the PHQ given. The PHQ-9 typically takes between 5 and 7 min for administration and scoring.

The original validation study of the PHQ-9 suggested a high internal reliability in primary care settings (α = 0.89) and obstetrics clinics (α = 0.86) that was comparable to that of the BDI-II [61]. The PHQ-9 demonstrated positive correlations with medical outcomes study short-form general health survey domains, including mental health (*r* = 0.73), general health perceptions (*r* = 0.55), social functioning (*r* = 0.52), role functioning (*r* = 0.43), physical functioning (*r* = 0.37), and bodily pain (*r* = 0.33) [61].

The diagnostic accuracy of the PHQ forms have primarily focused on the PHQ-9, with one meta-analysis concluding that inadequate research on the PHQ-2 was available to perform analyses [62]. Two different scoring methods have been examined: a simple sum score, and an algorithmic score. A recent individual participant data meta-analysis reported a sensitivity of 0.88 and specificity of 0.85 for a cut-off sum score of 10 or more compared to a semi-structured diagnostic interview [63], which is similar to a prior meta-analysis, where a cut-off of 10 or more had a sensitivity of 0.85 and specificity of 0.89 [64]. The algorithmic approach appeared to produce much greater specificity (0.94), at the cost of sensitivity (0.53), while the sum score was more balanced overall (specificity = 0.85, sensitivity = 0.77) [65].

Based on the reliability and variability of scores among those with depression [61], a change of three or four points is needed to be considered a reliable change in depressive symptom reporting (see Table 1). Sum scores greater than 10 on the PHQ-9 appreciably increase the probability of a depression diagnosis from 0.07 (per [37]) to 0.31, though alternative thresholds and scoring methods may be useful for specific program needs.

#### 2.2.5. Quick Inventory of Depressive Symptomatology

The quick inventory of depressive symptomatology (QIDS) is a 16-item self-report (QIDS-16_SR_) or clinician-rated report (QIDS-16_CR_). Both forms of the QIDS typically take between 5 and 7 min to administer. Unlike other scales reviewed for depression screening, the QIDS has primarily been used in research-settings, including most notably the Sequenced Treatment Alternatives to Relieve Depression (STAR*D) Study [66]. The QIDS is free to use and can be downloaded from http://ids-qids.org/ which includes additional resources for users. Unlike the other reviewed screening measures, the QIDS primarily aims to describe depressive symptom severity, rather than to aid in diagnosis; however, the QIDS’ website does include a table for converting between QIDS’ scores and scores on the Hamilton rating scale for depression. A meta-analysis of the QIDS’ psychometric properties ultimately concluded that more research on the scale’s validity, and in populations outside of the United States, is needed. These authors report a range of reported internal consistencies from α = 0.69 to α = 0.89 (self-report, though similar values for clinician-rating), and in their systematic review, they identified no studies that examined the test-retest reliability of either QIDS form. The scale appears to be unidimensional, and a meta-analytic correlation with other depression scales of *r* = 0.76 was found, suggesting good overall evidence for convergent validity [67].

#### 2.2.6. Summary of Depression Scales

The PHQ-9 and CES-D are two well-researched, brief, and freely available depression screening inventories. Compared to the PHQ-9, there are more studies among burn populations that have used the CES-D, making it perhaps the preferred option, given otherwise similar psychometric properties. In patients who are not able to respond to questionnaires, the HAM-D or QIDS may be preferred, as there are forms for clinicians to rate depressive symptoms based on observation, and the HAM-D has more supporting literature than the QIDS, making it the preferred scale in those cases. Finally, if there is interest in measuring change in depressive symptom severity over time, then the BDI-II is ideal. In cases where the cost of the BDI-II may limit its use, the PHQ-9 has sufficient data to compute at least a reliable change index. Some healthcare systems have implemented the PHQ as a general screener, so providers may also consider the relative benefit of using a PHQ when scores from pre-burn medical visits can be used for reference. Similarly, if administration time and staff burden are significant concerns, then use of the PHQ-2 might be considered.

### 2.3. Anxiety Scales

Prevalence of anxiety after burn injury varies depending on the burn severity, cause of burn, and type of anxiety disorder [38,68,69,70,71]. The presence of anxiety symptoms among burn patients is an important clinical factor to identify and treat. Anxiety is one of two factors (the other being alienation) predicting long-term psychological distress after burn injury [72]. Exposure to burns also appears to increase long-term risk for anxiety disorders. Assessing anxiety symptoms is, thus, clinically relevant to the management of the acute and long-term outcomes of burn patients.

#### 2.3.1. Beck Anxiety Inventory

Similarly to the BDI-II, the Beck anxiety inventory (BAI) can be obtained through Pearson (starter kits start at USD 98.40). The BAI is a 21-item, self-report screening and symptom severity questionnaire designed to measure subjective, somatic, and panic-related symptoms of anxiety, which can be administered in about 10 min. The scale is widely used and has been examined in a variety of settings, including in primary care [73], among family caregivers of children with cancer [74], older adults [75], veteran populations [76], and even as an outcome measure in a pediatric burn camp in Nicaragua [77]. A recent item response theory analysis of the BAI found that, while there was some evidence of differential item functioning, the scale is only minimally affected by sex, educational attainment, ethnicity, and testing language (English versus Spanish), such that the difference in scores between these groups is clinically negligible [78].

The robust validity evidence for the BAI is further supported by a systematic review and meta-analysis that found consistent convergent validity with other anxiety scales, as well as a stable two-factor model of the BAI across studies [79]. The meta-analytic internal consistency for the BAI was an impressive α = 0.91, with a test-retest reliability of 0.65 over a mean time of 6 weeks [79]. Combined with an aggregated standard deviation of 8.76 among non-clinical populations, a reliable change in BAI would correspond to about a 5 point shift in raw score (see Table 1). A single cut-off score for ruling in an anxiety disorder on the BAI was not reported; instead, studies reported varying cut-offs, ranging from 7 to 26, with four studies selecting a value of 16, and two studies selecting values of 7, 10, or 14 [79]. Areas under the curve, a global summary of diagnostic accuracy, where values of 1.00 reflect perfect accuracy and 0.50 reflect chance-level assignment, ranged from 0.63 to 0.77 [79].

#### 2.3.2. Hospital Anxiety and Depression Scale

The hospital anxiety and depression scale (HADS) may be an efficient option for clinicians wanting to generally summarize mood disorder symptoms, particularly given the significant overlap of depression and anxiety disorders. The HADS is a 14-item, self-report scale with seven items corresponding to a depression index, and the other seven to an anxiety index. Typical administration and scoring time is between 5 and 10 min. While the scales’ items can be obtained from the original publication [80], many translated versions are available by request from the Mapi Research Trust (https://eprovide.mapi-trust.org/instruments/hospital-anxiety-and-depression-scale).

Each item is scored on a 0–3 scale. Utilizing individual participant data, a meta-analysis found that the HADS had a sensitivity of 0.82 and specificity of 0.78 for depression, using a cut-off score of 7 or higher (specificity increases to 0.44 with sensitivity of 0.44 at a cut-off of 11 or more) [81]. These utility statistics were similar to those reported in two other meta-analyses of depression detection [82,83]. One of these meta-analyses also examined the HADS’ ability to detect anxiety disorders: sensitivity = 0.84 and specificity = 0.49, with the HADS’ total score (using all 14 items) outperforming the HADS’ anxiety index score; although, this was only among studies involving cancer and palliative care settings [83]. Overall, the HADS may be a useful general distress screener, but may not have the desired clinical utility for ruling in or out anxiety disorders among patients.

The HADS has been utilized in multiple studies involving burn patients, with varying results [36]. For example, one study reported that incidence of anxiety or depression as measured by the HADS did not differ between a burn and reference group, at either 12 or 24 months after injury; however, those who had higher levels of anxiety or depression on the HADS also had a poorer health-related quality of life [84]. In contrast, another study found that over a third (37%) of patients with less than 20% total body surface area burns reported elevated anxiety scores on the HADS two weeks after discharge from the burns unit [85]. This rate of elevated anxiety scores on the HADS is consistent with at least one other study [86]. 

#### 2.3.3. Generalized Anxiety Disorder Assessment

This seven-item, self-report scale of generalized anxiety is generally co-administered with the PHQ-9, and like the PHQ-9, it is freely accessible in a range of languages through https://www.phqscreeners.com/. The generalized anxiety disorder assessment, or GAD-7, also has a short form, like the PHQ-9, consisting of just two items, the GAD-2. Abbreviation of the GAD-7 may not be needed, as its administration and scoring time is generally less than 5 min. Considering its free-to-use nature and the number of translations available, the GAD-7 is commonly utilized as a screening measure in research, and as a result, it is also one of relatively few symptom inventories to have sufficient data to be examined in low to middle income countries, rather than just in industrialized, Western/European countries [87].

The GAD-7′s meta-analytic sensitivity for generalized anxiety disorder is 0.83, with a specificity of 0.84 using a cut-off score of 8 or higher (though the authors note that a similar performance is seen using cut-offs of 7 to 10) [88]. These results mean that a GAD-7 score greater than 8 increases the pre-test odds of having generalized anxiety disorder by about 5.2 times (see Table 1). The GAD-2, with a cut-off score of 3, produces fairly similar overall utility statistics: sensitivity = 0.76 and specificity = 0.81 [88]. The relatively simple structure and psychometrics of the GAD-7 and GAD-2 have also been replicated by more nuanced analyses, such as item response theory [89], providing strong overall evidence for criterion and construct validity. The reliability of the GAD-7 in a sample of 2149 patients from 15 primary care sites in 12 states was an impressive α = 0.92, and score variability was similar between patients with (standard deviation = 4.7) and without (standard deviation = 4.8) generalized anxiety disorder [90]. Using these results, a reliable change in GAD-7 scores is around 3 points (see Table 1). Utilizing the GAD-7 as a measure of anxiety symptom severity, pain was found to be positively related to anxiety scores, and anxiety and depression (per the PHQ-9) together partially mediated the relationships between pain and work functioning after burn injury [91].

#### 2.3.4. Summary of Anxiety Scales

As a brief, free-to-use, and well-researched scale, the GAD-7 is the preferred anxiety disorder screening tool; however, users should be cautious with its use, as it is intended to detect only generalized anxiety disorder, and not other anxiety-related disorders. When tracking of anxiety symptoms is desired, then the BAI is preferable, as it includes a greater range of scores and assesses more than just generalized anxiety symptoms. In burn settings, clinicians should consider the contributing role of pain, stress, and medications on somatic symptoms of anxiety (e.g., restlessness, difficulty concentrating, fatigue).

### 2.4. Trauma Scales

Given the absence of a clear association between burn severity and risk, general screening for trauma-related disorders is the best method for identifying potential clinical needs, and in cases where individuals remain connected to burn clinics for outpatient follow-up ongoing screening is warranted.

#### 2.4.1. Post-Traumatic Stress Disorder Checklist for DSM-5

The post-traumatic stress disorder checklist for DSM-5 (PCL-5) has been updated to be consistent with the PTSD diagnostic criteria in the most recent DSM-5 update [92,93]. The scale consists of 20 self-report items corresponding to each of the 20 PTSD symptoms in the DSM-5. Despite the relatively large number of items, the scale still typically takes less than 10 min to administer and score. It can be accessed free of cost, along with interpretation recommendations and references, through the United States Veterans Affairs (VA): https://www.ptsd.va.gov/professional/assessment/adult-sr/ptsd-checklist.asp. Initially developed through the VA services, the PCL has been widely used outside of its originally intended military populations. For example, the PCL-5 was recently validated among Chinese healthcare workers responding to the COVID-19 pandemic [94]. More relevant to this review, there has been one pilot study supporting the use of the PCL-5 among parents of children with burns [95], and a recent study utilized the PCL-5 to examine the prevalence of PTSD in burn patients and their family members [96]. Extensive research on the PCL-5′s psychometric properties is still lacking, though previous versions of the PCL demonstrated acceptable properties [97]. As per the VA, scores greater than 31 may be considered as evidence of probable PTSD, and changes of 5–10 points in scores may be considered a reliable change, while changes of 10–20 points may be thought of as clinically significant change, though this is based on previous research on the PCL-IV. Several translations of the PCL-5 have been developed by independent researchers including into Arabic, Kurdish dialects [98], German [99], Brazilian Portuguese [100], and French [101].

#### 2.4.2. Child PTSD Symptom Scale for DSM-5

The child PTSD symptom scale for DSM-5 (CPSS-5) utilizes a structure similar to that of the PCL-5, but was intended for children aged 8 to 18 [102]. There is a self-report scale (CPSS-5-SR) and an interview form (CPSS-5-I), and initial psychometric properties appear to be acceptable [102]. The internal consistency of both the interviewer version (α = 0.92) and self-report version (α = 0.92) are high, with stable test-retest reliabilities (*r* = 0.93 and *r* = 0.80 for the interviewer and self-report versions, respectively), though further research is needed [102].

#### 2.4.3. Clinician-Administered PTSD Scale for DSM-5

The clinician-administered PTSD scale for DSM-5 (CAPS-5) is endorsed by the United States’ VA as the gold standard in PTSD assessment. The scale is a 30-item structured interview, used to make either a current or lifetime diagnosis of PTSD, and it can be obtained through the United States’ VA (https://www.ptsd.va.gov/professional/assessment/adult-int/caps.asp). The CAPS-5 takes between 45 and 60 min to administer. As with the PCL-5, relatively little research using the CAPS-5 has been published. The initial validation study of the CAPS-5 revealed good inter-rater reliability (κ = 0.78–1.00), internal consistency (α = 0.88), test-retest reliability (ICC = 0.78), and positive convergent and divergent validity evidence; however, additional research in other populations and by other researchers is needed [103]. Training on the administration of the CAPS-5 is available through the United States’ VA in the form of a three-course curriculum. Several translations of the CAPS-5 exist with validations studies available for the Dutch [104], Brazilian Portuguese [105], and forthcoming German [106] versions.

#### 2.4.4. Primary Care PTSD Screen for DSM-5

As with the other PTSD screening forms reviewed so far, the primary care PTSD screen for DSM-5 (PC-PTSD-5) is a recently updated version of previous PC-PTSD scales, to align items to the new DSM-5 PTSD criteria. As such, there is less relevant research on the PC-PTSD-5 than on its previous iterations. The PC-PTSD-5 includes five yes/no, self-report items, intended to screen individuals for PTSD, which can usually be completed in less than 5 min. The current revision added an item to the previous PC-PTSD to reflect the new dimension of guilt or distorted sense of blame regarding the trauma in the DSM-5. As with the PCL-5 and CAPS-5, the PC-PTSD-5 can be obtained free of cost through the United States’ VA (https://www.ptsd.va.gov/professional/assessment/screens/pc-ptsd.asp). The initial validation study found that the PC-PTSD-5 was overall highly accurate in the identification of PTSD (area under the receiver operator characteristic curve = 0.94) and had an optimal sensitivity to PTSD when a cutoff of 3 was used (sensitivity = 0.95, specificity = 0.85) [107]. A more recent follow-up study broadly confirmed these initial findings of high overall accuracy (area under the receiver operator characteristic curve = 0.93) and acceptable utility statistics at a cutoff of 3 (sensitivity = 0.90, specificity = 0.80), but the authors specifically note that while a cutoff score of 4 was preferred for balancing false positives and negatives (sensitivity = 0.78, specificity = 0.91), a cutoff of 3 was better for women, as it dramatically reduced the number of false negatives [108]. While these studies suggest that the PC-PTSD-5 is an accurate, simple PTSD screener, it is important to note that both of these studies were in veteran populations and further generalization research is needed. The PC-PTSD-5 has been adapted for use among Chinese children [109], Chinese healthcare workers [110,111], and Korean adults [112].

#### 2.4.5. Injured Trauma Survivor Screen

The injured trauma survivor screen (ITSS) is a relatively recent scale designed specifically as a hospital-administered screening measure for PTSD that was developed and normed on patients admitted to the trauma service of a Level 1 Trauma Center [113]. The scale consists of 9 yes/no self-report questions selected from a larger initial pool of items, based on how predictive they were for PTSD and a major depressive episode at one-month post-injury [113]. As a result, the scale is specifically designed with predictive validity in mind. The scale takes about 5 min to administer. Using a cut-off score of 2 for the PTSD subscale, the sensitivity for PTSD one month after injury was 0.75, while the specificity was 0.94 [113]. Using the same cut-off score, the sensitivity was 0.85 and specificity was 0.67 for PTSD at six-months after injury [114]. A more recent study predicting PTSD at one-month after injury, however, found less impressive utility statistics: sensitivity = 0.72, specificity = 0.60 [115]. Additional research using the ITSS is needed.

#### 2.4.6. Summary of Trauma Scales

For the purposes of screening for PTSD, the PC-PTSD-5 is well-validated and brief, with good overall psychometric performance. In cases where there is reasonable a priori concern for PTSD, forgoing the PC-PTSD-5 and using the PCL-5 may be more sensitive and better characterize PTSD severity. When a definitive diagnosis of PTSD is needed or deemed useful, the CAMS-5 is the gold-standard, but the required training and required clinician administration make this burdensome to use, particularly for burn patients in the acute phase of recovery.

### 2.5. Suicide Scales

Reliable methods for the prediction of suicidal behavior are elusive and reviews of existing studies that aimed to predict suicide have concluded that efforts may have limited effectiveness at best, or create harmful overconfidence in individual risk at worst [116,117,118,119,120,121]. Complicating the prediction of suicide risk are the risk factors that are relatively common in both general and clinical populations, weak associations between ideation and attempt, low base rates of attempt, and complicated inter- and intra-individual differences in risk and protective factors [119,120,122]. Fortunately, more recent research has confirmed the efficacy of proactive screening methods for identifying suicide risk among vulnerable populations [122,123]. Importantly, screening for depression, even on scales where there is a suicidal ideation item (e.g., the PHQ-9 or BDI-II), is inadequate, as a large number of individuals who would screen positive on a suicide risk screening do not necessarily endorse high levels of depression [124,125]. 

#### 2.5.1. Ask Suicide-Screening Questions

The ask suicide-screening questions (ASQ) tool is a clinician-administered, four-question screening instrument that was developed through a multisite National Institute of Mental Health study [126]. The scale can take less than a minute to administer. Since the initial validation of this study, the ASQ has been examined and validated in adult medical patients as well [127]. The tool has also been studied under both selective and universal screening protocols: a positive ASQ was associated with a 6.8 hazard ratio (universal screening) and 4.8 hazard ratio (selective screening) for subsequent emergency department visits with suicide-related presenting problems and death by suicide [128]. In a universal screening study that utilized the ASQ, 2.9% of hospital-wide encounters of patients between ages 10 and 17 screened positive for elevated suicide risk [129]. Compared to the suicidal ideation questionnaire, the 4-item ASQ has demonstrated negative predictive probabilities of about 0.99 in multiple studies [129,130,131,132]. Note that the low base rate of suicide attempts makes the positive predictive probabilities of suicide screening tools less informative, and readers should keep in mind that base rates of suicide among burn patients may be different than in the general medical or psychiatric populations where most of these scales have been examined. Other psychometric properties, aside from utility statistics, have not been studied, likely due to the scale’s short length. The tool, educational/training resources, and its 13 translations are available through the National Institute of Mental Health (https://www.nimh.nih.gov/research/research-conducted-at-nimh/asq-toolkit-materials). 

#### 2.5.2. Columbia-Suicide Severity Rating Scale

The Columbia suicide severity rating scale (C-SSRS) is available for free through The Columbia Lighthouse Project (https://cssrs.columbia.edu/the-columbia-scale-c-ssrs). The Columbia Lighthouse Project provides multiple versions of the C-SSRS, including for lifetime, recent, and since last visit screening of suicidal ideation and behavior, and they provide targeted resources and forms for multiple settings (e.g., schools, corrections, healthcare, etc.). Administration time will vary based on the version used and patient history, but typical administration is about 5 min. Additionally provided through these resources are over 140 translations of the C-SSRS scales, access to training resources, and an extensive supporting evidence document. The C-SSRS has been widely adapted as a component of standard suicide screening protocols [130], though its qualifications as a gold-standard scale have been questioned [133]. Utilizing the criterion of any C-SSRS item being endorsed as reflecting a positive screen, one study found that, of the 3712 trauma patients administered with the C-SSRS, 4% screened positive, and a positive screen was associated with being White, speaking English as a primary language, and having insurance coverage (no differences in age, sex, marital status, or type of trauma) [134].

#### 2.5.3. Beck Hopelessness Scale

As with the other Beck scales, the Beck hopelessness scale (BHS) can be obtained through Pearson (starter kits beginning at USD 98.40). The BHS is a 20-item, true/false questionnaire that has been used as an adjunct scale to describe depressive symptoms and also to predict suicidality. Administration typically takes between 5 and 10 min. Internal consistency estimates for the BHS have ranged from 0.65 to 0.93 with lower reliability being observed in non-clinical samples [131]. Based on reliability and score variation data from non-clinical samples, a reliable change in BHS scores is between 6 and 7 points (see Table 1). Using a cut-off score of 9 or greater, a meta-analysis of the BHS’ predictive accuracy for suicide (diagnostic odds ratio = 3.39, sensitivity = 0.80, specificity = 0.42) and non-fatal self-harm was reasonable (diagnostic odds ratio = 2.27, sensitivity = 0.78, specificity = 0.42) [132].

#### 2.5.4. Summary of Suicide Scales

Operationalized and systematic screening of suicidal ideation is recommended as a universal practice. Both the ASQ and C-SSRS are well-validated and come with a variety of supporting resources to assist in safety planning and triaging of concern. The ASQ is simple to utilize and brief, while some of the C-SSRS scales (e.g., the lifetime risk scale) can require some prior training and practice to become comfortable with the scale. While not as useful as a suicide screening scale, the BHS can provide useful information to collect for referral resources, as individuals who are endorsing high levels of hopelessness may benefit from more targeted mental/behavioral health, supportive, or pastoral care.

### 2.6. Delirium Screener

Burn patients who develop delirium have longer durations of stay (including after adjustment for age and burn size), higher rates of psychiatry consultations, and greater mortality [23,24,25]. Considering the significant effect of delirium on outcomes and the overall susceptibility of burn patients to developing delirium, it is somewhat reassuring that about 70% of clinicians report assessing burn intensive care unit patients for delirium, though more than half (57.5%) do not utilize a specific scoring system in this assessment [135].

#### 2.6.1. Confusion Assessment Method

Likely the most commonly used delirium assessment, the confusion assessment method (CAM) is a nine-item, clinician-rated measure intended to detect fluctuations in cognitive status typical of delirium [136]. An alternative version, the CAM-ICU, is available for use in non-verbal or ventilated patients. Meta-analyses of the CAM’s diagnostic algorithm have generally found that the measure is fairly accurate: sensitivities = 0.82–0.94, specificities = 0.89–0.99 [137,138]. Classification rates of delirium are similar among meta-analyses of the CAM-ICU: sensitivities = 0.80–0.85, specificities = 0.95–0.98 [137,139,140,141]. The relatively low sensitivity compared to the high specificity of the CAM-ICU has been cited as a clinical limitation, as many individuals with delirium may be missed by the measure [142]. The sensitivity with the CAM may be improved when individual baseline risk factors are also considered in the context of a questionable or negative CAM score. The CAM has also been translated and validated into 10 different languages [138].

#### 2.6.2. Intensive Care Delirium Screening Checklist

The intensive care delirium screening checklist (ICDSC) was developed to address the language dependency of many other delirium screening scales, including the CAM [143]. The checklist consists of eight items corresponding to the DSM’s criteria for delirium, with the intention that these items can be scored based on observation during discrete assessment periods, and the checklist and administration rules can be found in the original validation study [143]. The ICDSC performed favorably in its initial validation, with sensitivity of 0.99 and specificity of 0.64 at a cutoff of 4, and a 94% agreement rating between nurses and physicians [143]. These initial results have largely been replicated by other studies, with cutoffs ranging from 3 to 5 producing good diagnostic accuracy, routinely high inter-rater agreement, and positive feedback from staff on ease of use [144,145,146]. Some studies have advocated for greater emphasis on the sensitivity of delirium screening with the ICDSC [147], and a lower cutoff of 3 has been found to produce sensitivities closer to those observed in the initial validation study [145,147]. Given the variability in utility statistics across studies, additional research is needed to identify an ideal cut-point for the ICDSC. Comparisons of the CAM-ICU to ICDSC have found them to have high levels of agreement and similar diagnostic validity [148,149,150]. Multiple translations of the ICDSC have been examined, including Italian [151], Swedish [152], Persian [153], Turkish [154], and Japanese [149]. One study recently examined the use of the ICDSC among burn patients in a mobile-based application that included the checklist, educational material about delirium, and patient data [155]. Using this application, the authors found that the diagnostic agreement of delirium between nurses and physiatrists was higher than for those who did not use the application, suggesting that this integration of tools and educational material can help reduce misdiagnosis due to inexperience, lack of delirium assessment training, or varying familiarity with the heterogeneity of delirium presentations [155].

#### 2.6.3. Summary of Delirium Scales

Both the CAM(-ICU) and ICDSC are well-known and familiar delirium screening tools. In hospital systems, selection of a scale that is given on other floors may help with effective screening of delirium, as it reduces the risk that staff trained on one instrument fail to correctly utilize another.

### 2.7. General Health/Psychopathology Screeners

With improvements in mortality, greater emphasis has shifted to morbidity [156]. The patient-reported outcomes measurement information system (PROMIS) and the brief version of the burn specific health scale (BSHS-B) are two methods for capturing the multidimensional nature of the sequelae of a burn injury. In conjunction with quality of life measures, these scales provide important information regarding burn-specific health status [157]. 

#### 2.7.1. Patient-Reported Outcomes Measurement Information System

The patient-reported outcomes measurement information system (PROMIS) is a set of measures evaluating and monitoring physical, mental, and social health in adults and children. The physical health profile domains assess fatigue, mobility, pain intensity, pain interference, upper extremity function. The additional PROMIS physical health domains assess asthma impact, pain behavior, pain quality, physical activity, physical stress experiences, sleep, and strength impact. The mental health profile domains assess anxiety and depressive symptoms. Additional mental health domains include anger/irritability, cognitive function, engagement, life satisfaction, meaning and purpose, positive affect, psychological stress experiences, and self-regulation). The social heath domain assesses peer relationships. The additional social health domains assess family relationships and social relationships. 

The PROMIS is self-administered via paper, computer (REDCap, Epic, Assessment Center Application Programing Interface, and other software platforms) or with an application (PROMIS iPad App, NIH Toolbox^®^ iPad App). There are several PROMIS health profiles, including the PROMIS-29, PROMIS-43, and PROMIS-57. If respondents are unable to respond themselves, due to cognitive impairment or communication deficits or disease burden, a proxy can answer on their behalf. PROMIS parent proxy forms are available for children ages 5–17, and there are self-report measures validated with children aged 8–17. There is moderate to good agreement in the pediatric PROMIS depression, physical function, peer relationships, pain interference, and anger scales with pediatric burn survivors ages 8+ and their proxy caregivers, suggesting that a proxy-report alone, as a surrogate, should only be considered when the pediatric burn survivor is not able to respond him- or herself [158]. PDFs of the measures are publicly available at https://www.healthmeasures.net/explore-measurement-systems/promis at no cost, in English and in Spanish. There are 4–10 items on short forms, and computer adaptive tests administer 4–12 items, depending on the respondent’s previous answers. Responses can be scored on the HealthMeasures Scoring service (https://www.assessmentcenter.net/ac_scoringservice) or using a data collection tool that automatically calculates scores (e.g., Assessment Center, REDCap auto-score). 

The PROMIS profile in several forms has been validated in numerous studies with different adult and pediatric patient populations and characteristics. The PROMIS-29 profile was recently validated for burn survivors 18+. The PROMIS-29 domains demonstrated high internal consistency (Cronbach’s *α* = 0.87–0.97). All domains except sleep (>0.85) were high (>0.9). A large part of the respondents did not report any symptoms in the domains of depression, pain, anxiety, and fatigue. There was a large ceiling effect on social roles (39.7%) and physical function (43.3%). The reliability of extreme scores and ceiling effects could potentially be improved by administering PROMIS-43, which includes six items per domain [159]. A validation study has not been completed for pediatric burn survivors. 

#### 2.7.2. Brief Version of the Burn Specific Health Scale

The brief version of the burn specific health scale (BSHS-B) was developed to assess different dimensions of health for burn survivors, as a shorter alternative to the abbreviated burn specific health scale (BSHS-A) and the burn specific health scale revised (BSHS-R). The scale includes a three-factor structure containing nine domains: affect and relations (interpersonal relationships, affect, sexuality), function (simple abilities, hand function), and skin involvement (heat sensitivity, treatment regimens, body image). The work subscale is considered a separate outcome domain. The nine domains are well separated, and factor intercorrelations range from 0.11 to 5.6. The scale demonstrated good internal consistency (Cronbach’s *α* = 0.75–0.93) and concurrent validity when compared with the BSHS-A and the BSHS-R [160]. The scale takes about 10–15 min to complete, has 40 items on a 5-point scale ranging from 0 (extremely) to 4 (not at all), and mean scores are calculated for the domains. Scores range from 0 to 40. A higher BSHS-B score indicates fewer problems. The BSHS-B is available in English and Swedish and is available in the appendix of the article by Kildal and colleagues [160]. The BSHS-B has been translated into several languages, including Korean [161], Chinese (ACV BSHS-B) [162], Portuguese (BSHS-B-Br) [163], German [164], Hebrew [165], Italian [166], Persian [167], Polish [168], and Turkish [169].

### 2.8. Body Image

#### Satisfaction with Appearance Scale

Burn survivors may experience negative body image due to changes in their appearance resulting from their injuries. Identifying burn survivors who experience negative body image may facilitate connection to appropriate clinical resources. The self-administered satisfaction with appearance scale (SWAP) assesses the subjective appraisal and social-behavioral components of body image, specifically among burn survivors. The scale has 14 items on a 7-point scale, ranging from 1 (strongly disagree) to 7 (strongly agree). The total score is calculated by subtracting one from each item and then totaling the answers, with questions 4–11 reversed score, for a total score range from 0 to 84. Higher scores indicate greater dissatisfaction with appearance and worse body image. The scale is offered in English and is available in the appendix of the article by Lawrence and colleagues [170]. The SWAP demonstrated good internal consistency (α = 0.87) and good convergent–discriminate validity. The SWAP was correlated with the physical appearance state and trait anxiety scale (PASTAS, [171]; (*r =* 0.63, *p <* 0.01) and demonstrated good discriminant validity, as the SWAP was not related to the measures of either physical functioning (*r =* −0.03, ns) or bodily pain (*r =* −0.07, ns) of the SF-36. Strengths of the scale include brevity, specificity, and ease of administration. The scale has been translated into several languages, including Korean [172], Brazilian [173], Swedish (SWAP-Swe) [174], and Urdu [175].

### 2.9. Community Integration

Community integration is the individual’s ability to be active in the community, at home, and the ability to participate in leisure activities, work, school, or volunteering. Assessment of community integration is an important component of identifying burn survivors who are experiencing difficulties with post-injury adjustment.

#### Community Integration Questionnaire

The 15-item community integration questionnaire (CIQ) measures home integration (score 0–10), social integration (score 0–12), and productivity (score 0–7), scored by subject self-report [176]. It can be self-administered or by a clinician and takes about 15 min to complete. High scores indicate greater independence and community integration. The scale is available from the Center for Outcome Measurement in Brain Injury (www.tbims.org/combi/ciq/index.html). The measure has demonstrated good reliability and validity for adults 18+ (see review paper by Dijkers) [177,178], also see the tbims website for a summary of clinimetric properties of the scale since 1997). The CIQ is considered to be at a 6–8th grade reading level and has been translated into several languages. 

A modified CIQ scale (CIQ-13) demonstrated validity and reliability as a two-factor model for burn survivors: self-family care in the home, and social integration outside the home [179]. The CIQ-13 does not include question 4 (Who usually cares for children in your home) or question 11 (Do you have a best friend with whom you confide?) from the original CIQ. The CIQ-13 demonstrated acceptable internal consistency (α = 0.80 for Factor 1, 0.77 for Factor 2, and 0.79 for the test as a whole).

### 2.10. Pain Assessment

Pain is a subjective experience, and often the patient’s report is accepted as the most valid measure of pain. Pain is often simply described using adjectives such as ‘none, mild, moderate, severe’ or ‘no pain, mild, discomforting, distressing, horrible, or excruciating pain’ [180]. Pain is underassessed and undermanaged in preverbal children and in children with cognitive impairment [181]. Measuring children’s pain using physiologic indicators (e.g., heart rate, respiratory rate, and blood pressure) is unreliable when a burn injury is present [182]. A variety of pain measurement scales have been used with adult and pediatric burn patients, but there have been no studies validating the scales for use with burn survivors.

#### 2.10.1. Numeric Rating Scale and Visual Analog Scale

Most burn units use the numeric rating scale (NRS) in adults [27]. The NRS is a horizontal line with an 11-point numeric range. It is labeled zero to ten, with zero indicating ‘no pain’ and ten indicating ‘worst imaginable pain’. The visual analog scale (VAS) includes graphic images in the range instead and is also commonly used. The benefits of using these scales include their brevity and their utility among diverse patient populations. They are valid and reliable for rating pain intensity and can be administered verbally and in writing. These scales are not able to capture other factors such as past pain experiences and are limited to the assessment of pain during a specific time period. The NRS and VAS have been validated in numerous studies with different adult patient populations and characteristics. The NRS has been studied in children aged 8 and older [183], and the VAS has demonstrated good acceptability, responsibility, and validity for most children aged 8 and older [184]. Use of NRS is cautioned in younger children and children with cognitive impairment, due to numeracy ability [184]. 

#### 2.10.2. Faces, Legs, Activity, Cry, Consolability Scale

The faces, legs, activity, cry, consolability scale (FLACC) is an observer rating scale most widely used to assess pain in preverbal children, beginning at 2 months old [180,185], and is available in several languages. Each of the five categories are scored from 0–2, with a total score between 0 and 10. For patients who are awake, children are observed for at least 2–5 min, while patients who are asleep should be observed for 5 min or longer. The FLACC demonstrated validity and reliability for preverbal children [185] and children with cognitive impairment [186].

#### 2.10.3. Wong–Baker FACES Pain Scale and the OUCHER Scale

Preschool children are able to complete simple self-report pain scales. Two common scales used are the Wong–Baker FACES pain scale and the OUCHER scale. Both scales appear to be equally reliable and valid, and previous research has suggested the consistent use of one or the other is most important [180]. The Wong–Baker FACES rating scale includes six faces that vary from smiling to grimacing to demonstrate how a person might be feeling. The scale is available at https://wongbakerfaces.org. It is indicated for people aged 3 and older and is currently available in over 60 languages. Benefits of using the Wong–Baker scale are that it is inexpensive and easy to use. Limitations include that some children may not cry with pain, preschool aged children may have the tendency to select faces at extremes of the scale [187], children may be embarrassed to choose a face with tears, and it assesses the intensity of pain, not the pain location, affective and behavioral components of pain, or impact on daily functioning. The Wong–Baker FACES pain scale has demonstrated good validity and reliability [188].

The OUCHER scale is a number scale for older children and a picture scale for younger children. The OUCHER is available at www.oucher.org with brief instructions available in English, Spanish, Dutch, and Chinese. The OUCHER scale has adequate validity and reliability [189]. Children who demonstrate numeracy can use the numeric scale. The picture OUCHER scale is unique, in that it shows a child of a different sex and race, to allow the child to relate to the pictures more easily [190,191]. Disadvantages of the OUCHER are that it can be difficult to use by younger children aged 3 to 7 years old, the photos are of acute rather than chronic pain, it may be more expensive to reproduce as it requires the printing of color photographs, and laminated scales need to be disinfected between patients. It may be helpful to allow the child to decide which pain measurement tool he or she prefers. In a study measuring pain in Black children, the FACES and Black OUCHER scales were found to be valid and reliable tools, and the FACES was the most preferred scale [192]. Adults with language barriers may also benefit from using picture scales. 

#### 2.10.4. Pain Assessment in Advanced Dementia Scale

Pain assessment for individuals with advanced dementia can be challenging. The pain assessment in advanced dementia scale (PAINAD) is a five-item observational tool with scores that range from 0 to 10 (based on a scale of 0 to 2 for each item) and is available in multiple languages. The PAIDAD takes approximately 5 min to complete. The scale demonstrated adequate levels of interrater reliability and has satisfactory reliability, but internal consistency was generally lower than the desired 0.70 (Cronbach’s α ranged from 0.30–0.80 [193]); however, a factor analysis demonstrated one underlying construct. The PAINAD and the discomfort scale-dementia of Alzheimer type (DS-DAT) were significantly correlated. The PAINAD detected differences in scores prior to and after receiving a pain medication, indicating that it is a simple, valid, and reliability instrument for measuring pain in noncommunicative patients [193].

### 2.11. Pruritus Assessments

Pruritus, or itching, is a multidimensional and subjective experience that is very common and unpleasant after a burn injury, and unidimensional methods to assess itching either only assess scratching or assess intensity, without acknowledging the effects on quality of life.

#### 2.11.1. 5-D Itch Scale

The 5-D itch scale was developed for adults as a brief instrument to assess the multidimensional nature of itching: degree, duration, direction, disability, and distribution. The duration, degree, and direction domains have one item, the disability domain has four items, and these questions are measured on a five-point Likert scale. The distribution domain has 16 potential areas of itch. Responses for the first four domains are scored on a 1–5 scale, and the highest score from the disability domain question is used. For the distribution question, a total of 0–2 sites equates to 1 point, 3–5 to 2 points, 6–10 for 3 points, 11–13 for 4 points, and 5–25 for 5 points. The score ranges from 5 to 25 points. The scale takes less than 5 min to complete. The validity of the measure is demonstrated by strong correlations, with a visual analogue score at baseline (0.272, *p* < 0.0001), at a 3-day repeat (*r* = 0.868, *p* < 0.001), and at a 6-week follow up (*r* = 0.982, *p* < 0.001). The measure demonstrates very high test-retest reliability and good ability to detect changes over time. The internal consistency (Cronbach’s α) was 0.734, and the agreement between repeated measures (intraclass correlation coefficient, ICC) was 0.96, 95% CI [0.92–0.98]. The 5-D was able to detect changes in pruritus at a 6-week follow up (*p* < 0.001). The 5-D itch scale is available in the original published article by Elman, Hynan, Gabriel, and Mayo [194]. The 5-D itch scale has been translated into several languages, including Arabic, Japanese, Thai, Spanish, Malay, Portuguese, and French.

#### 2.11.2. 4-D Itch Scale

The 4-D itch scale was modified from the 5-D itch scale because the distribution of itching associated with burns was different from other medical conditions, as itching only occurred at the site of the burn. The 4-D itch scale contains all questions, except the last question regarding distribution. The 4-D also differs from the 5-D in scoring. The 4-D adds the single-item scores from duration, degree, and direction and the four items from the disability domain. The scores ranged from 5 to 35. The internal consistency (Cronbach’s α) was 0.82. One item (direction) had a corrected item-total score correlation of 0.28, indicating the psychometric properties may be improved by removing this item [195]. These results support the use of the 4-D scale for burn survivors, in the context of the limitations discussed.

#### 2.11.3. Itch Man Scale

Scales frequently used for adults such as the visual analog scale (VAS) or the 5-D itch scale (Elman et al., 2010) can be difficult for children to complete, due to reading level and difficulty understanding the questions. The itch man scale is a self-report scale created by Patricia Blakeney, PhD, and Janet Marvin, RN, that has drawings that signify discomfort associated with itching [196]. It is copyrighted by Shriners Hospital for Children. The drawing has five pictures depicting an itch man. His facial expressions and amount of area covered with red dots correspond to the itching intensity. It has a five-point Likert scale rating the intensity of itching, with 0 representing itching, and 4 indicating immense itching and distractibility. A child six and older can complete the scale independently, while parents or providers can answer for a younger child. In a study with pediatric burn patients ages 6 and older, the scale had a high correlation between two observers of the same child (*r =* 0.912, *p* < 0.01). The itch man scale correlated significantly with the VAS (*rs* > 0.772, *p* < 0.01). These results indicate the itch man scale is a reliable and valid method to assess itching in pediatric burn survivors [197].

### 2.12. Quality of Life Measures

Modern medicine traditionally assesses changes in patients by focusing on clinical or laboratory tests. While this provides important information about a disease, social and personal contexts also significantly affect the course of medical treatment. Quality of life (QoL) measures ensures treatment focuses on the patient, rather than the disease [198]. Data about QoL can help providers tailor treatments to provide holistic and patient-centered care.

#### 2.12.1. Medical Outcomes Study Short Form and SF-36v2^®^ Health Survey

The medical outcomes study short form (36; SF-36) health survey is a 36-item self-report questionnaire of the patient’s health, for adults 18 years of age or older. It has eight scaled scores, in the following domains: vitality, physical functioning, bodily pain, general health perceptions, physical role functioning, emotional role functioning, social role functioning, and mental health, with good to excellent internal consistency across all domains (Cronbach’s α = 0.81–0.95; [199]). The lower the score on the measure, the greater the disability, with 0 equivalent to maximum disability, and 100 equivalent to no disability. The SF-36 has been extensively used to evaluate individual patients’ health status, the cost-effectiveness of a treatment, and for monitoring and comparing disease burden. It was not developed for a specific disease, age, or treatment, and can be administered in a variety of settings. The original SF-36 (also referred to as RAND-36; SF-36 version 1) questionnaire is available free of charge from the RAND Corporation (http://www.rand.org/health/surveys_tools/mos/mos_core_36item_survey.html). The questionnaire was modified in 1996 with regards to item formulations and possible answers, which resulted in the commercial version of the SF-36 (also known as SF-36 v2.0; SF-36v2^®^). 

The revised SF-36 can be obtained from QualityMetric (http://www.qualitymetric.com). Pricing is contingent on the number of scores the researcher needs to calculate and whether the survey is used in a nonprofit or commercial setting. The SF-36v2^®^ is self- or interview-administered via paper, online, or interactive voice response (IVR) via telephone. The survey takes about 7–10 min to complete, but cognitive impairment, physical impairment, and depression could result in longer completion times [200]. There are over 213 translations available. The reading level of the SF-36v2^®^ is 6.4 (easy) according to the Flesh–Kincaid and Flesch reading ease formulas. There was notable variation in item readability, and the scores ranged from 2.2 to 12.0 (very easy to difficult; [201]). The SF-36v2^®^ is available in two forms: a standard form (4-week recall period) and acute form (1-week recall period). The 4-week recall period is used when the survey will be administered only once, or when there is at least a difference of 4 weeks between the initial and subsequent administration. The acute form is used when there is more frequent administration or when the health status is expected to rapidly change [200]. In a study comparing the validity of the SF-36^®^ in burn survivors to the burns specific health scale (BSHS), the SF-36v2^®^ domains showed significant associations at all time points (*r* = 0.37–0.76, *p* < 0.002), indicating the SF-36^®^ is a valid measure of recovery of quality of life in the burn patient population [202].

#### 2.12.2. SF-12v2^®^ Health Survey

The SF-12v2^®^ Health Survey is a shorter version of the SF-36v2^®^ and has 12 questions to measure health and well-being for adults aged 18 and older. The SF-12 measures the same eight health domains as the SF-36, with two summary components (mental component summary and physical component summary), has a standard form (4-week recall period) and acute form (1-week recall period), the same types of administration (e.g., paper, online, IVR), and scoring on a 0–100 scale, with lower scores indicating more disability. The reading level of the SF-12 is at a 6th grade level. The major benefit of the SF-12v2^®^ health survey is that it takes 2–3 min to complete. The SF-12v2^®^ health survey is also available for purchase from QualityMetric, and costs differ, similarly to the SF-36v2^®^ [200]. There are currently 205 translations available. Scores on the SF-12v2^®^ health survey have been found to be nearly identical to the SF-36v2^®^ [203]. The SF-12v2^®^ health survey has been validated for many other health conditions, but no studies have investigated the validity of the SF-12 for measurement of the temporal recovery of QoL outcomes in burn survivors.

#### 2.12.3. SF-10™ Health Survey for Children

The SF-10™ health survey for children is a 10-item parent completed survey for children aged 5–18. The SF-10™ health survey for children can be completed online, on paper, or via an interview and takes 2–3 min to complete. It is available for purchase from Quality Metric, and the costs differs similarly to the other Short Form surveys. There are currently 170 translations. While the SF-10™ health survey for children has been validated for other health conditions, no studies have investigated the validity for pediatric burn patients. 

#### 2.12.4. Life Impact Burn Recovery Evaluation Profile

The life impact burn recovery evaluation (LIBRE) profile assesses social participation after a burn injury. It measures six domains of social participation: relationships with family and friends, social interactions, social activities, work and employment, romantic relationships, and sexual relationships. The LIBRE-Profile has been studied in adults 18 years of age or older. Scores on the LIBRE profile provide clinicians with information that can help inform treatment plans and evaluate programs targeting social participation after serious burn injury [204].

The LIBRE profile can be administered via a computerized adaptive test (LIBRE Profile-CAT) or fixed short forms (LIBRE Profile-SF). The LIBRE Profile-CAT is responsive to the respondent’s answers in real time. For example, if the respondent has difficulty with the situation in the question, the next question will ask about a less difficult situation. The LIBRE Profile-CAT will administer questions until a level of precision is reached. Consequently, the LIBRE-Profile CAT administers 6–10 questions per scale, while the LIBRE Profile-SF administers 10 questions per scale. Respondents answer between 18–60 questions when completing the LIBRE Profile-CAT, and 30 to 60 questions when completing the LIBRE Profile-SF. The survey takes 15–30 min to complete. The LIBRE profile has repeatability coefficients from 7.31–9.27 and SEMs ranging from 2.62–3.39 for scales. The correlations between the scales and legacy measures are significant for both convergent and divergent validity [204]. The LIBRE profile is currently not publicly available. Interested parties can contact BostonHarvardBIMS@partners.org to obtain the LIBRE Profile. The LIBRE profile is currently being translated into multiple languages. 

An additional scale measuring social participation after a burn injury for young children is currently being developed, called the Preschool_1–5_ life impact burn recovery evaluation (LIBRE) profile. This age group is unable to participate in traditional paper-based instruments and differs in how they are affected by burns compared to adults. Research is currently underway investigating four item pools: (1) communication and language development, (2) physical functioning, (3) psychological functioning, and (4) social functioning for preschool-aged children. These domains are currently being field-tested to calibrate and validate the Preschool_1–5_ LIBRE computer adaptive test (LIBRE-CAT) profile [205]. A school-age version is also in development that will investigate the following domains: (1) physical functioning, (2) psychological functioning, and (3) family and social functioning [206].

## 3. Conclusions

This review provides a brief overview of the psychological and psychosocial challenges that burn survivors may experience and scales for assessing these factors. The instruments above give providers valuable information to make informed decisions about potential health needs of the individual burn survivor. However, it is not recommended to use each of these scales described. Administering, completing, and scoring multiple assessments can be burdensome for both clinicians and patients. If the patient is unwilling or unable to complete the assessment accurately, the tool becomes ineffective. Discussing the value of the instruments (e.g., guiding treatment or in context of patient goals), allowing patients to choose when to complete the instruments (e.g., before an appointment, in the evening if hospitalized), and providing different options of how they can fill out assessments (e.g., pen and paper vs. online) can improve willingness to accurately complete the instruments. The following recommendations may be helpful in considering when and what instruments to use [206]:

- Select an instrument that is meaningful and practical.
- Dedicate time and training to effectively integrating the assessments into the practice and understand that workflows may be interrupted.
- Identify how to analyze and prepare data so the healthcare team can easily view results.
- Having referral sources and resources ready and available to intervene on results.

To assess for psychiatric comorbidities, there are several free options to use with similar characteristics that are brief and easy to complete. These scales are particularly helpful in guiding referrals and treatment options, when clinicians are familiar with the scales and understand how the burn injury may be affecting responses. For example, burn survivors may be sleeping 10+ ha day, moving much more slowly, feeling like they have no energy, and have poor appetite. However, for many patients, these symptoms may be caused by pain medications or from the burn injury itself. This information is important, as scale scores may be higher due to endorsement of these items, but do not necessarily represent symptoms of a mental health disorder. The focus of using these scales, therefore, should focus on identifying individuals who may need additional support, consulting/planning for these needs, and referral to or consulting mental health professionals as needed. Mental health professionals can help provide insight into whether the symptoms are in context of an adjustment reaction, medical illness, or whether these symptoms are exposing an existing, underlying mood disorder or triggering a recurrence of a preexisting mood disorder. Overall, there is no ‘right’ scale to choose. Clinicians should choose scales they are comfortable with and knowledgeable about using, and use the scale that best examines what the clinician is trying to find (e.g., rate severity vs. finding disorders, detecting adjustment vs. depression). 

With the caveat that scale selection and utilization should be determined by clinicians for their specific needs and setting, some general recommendations can be made. First, routine or even universal screening for depression, anxiety, and suicide at admission and discharge may be considered, due to either the base rate of these concerns in burn populations or due to safety and because of the treatment and disposition implications of concerns. For this purpose, brief scales such as the PHQ-2, GAD-7, and ASQ may be preferred to reduce staff and patient burden. Clinicians may also consider the utility of quality of life and body image scales at discharge, when patients are expected to follow-up in outpatient clinics, as these general concerns may change over time as treatment focus shifts from acute stabilization and survival to long-term recovery and coping, making the baseline scores at discharge a potentially valuable resource for longer-term care. Second, consideration, plans, and resources should be developed in advance of any implementation of routine screening. Determination of available consultancy services (e.g., psychology, psychiatry, pastoral care, palliative care) or community resources should be made, so that positive screening can lead to immediate referral and continuity of care. Similarly, step-up screening for individuals who are positive on briefer scales should be considered in advance. For example, someone who scores positively on the PHQ-2 may be administered the PHQ-9 next. Similarly, if a score is elevated on the GAD-7, then administration of the PC-PTSD may be warranted, to examine whether anxiety symptoms are elevated due to an underlying acute/post-traumatic stress disorder. Third, the integration of mental health scales with patient recovery indicators is important. Multiple studies discussed in this review have found an important mediating role of depression and anxiety in pain, quality of life, and distress. The implication of this is that the management and treatment of these mental health factors should be considered as part of the medical treatment of burn outcomes and disposition planning. Tracking depressive symptoms and pain, for example, may help to inform about pain management changes to medications, and integration of psychology or psychiatry (particularly those with familiarity with chronic pain or interdisciplinary pain management) may be a vital resource for the pain management plan. Finally, integration of any selected scales with medical records is highly recommended. Allowing staff to directly enter symptom scale responses or scores into the medical record can help to reduce burdens and recording errors. At the same time, having the ability to visualize structured symptom reporting over time can help clinicians make informed decisions about treatment changes. For example, in patients for whom pain management is a limiting factor, the ability to track pain symptoms over time and correlate any changes in scores to changes in treatment is invaluable for making evidence-based care decisions and setting clear, measurable, and attainable discharge goals.

There are several scales that have been created for the burn population and the unique challenges they face. There are several other scales that have been validated for the general population and other medical conditions. One concern and limitation in the literature at present is the relative dearth of studies validating scales in burn populations, as it is more common to see studies involving burn patients utilize scales validated in other populations. This presents opportunities for further research to validate these scales among burn survivors, to ascertain and better understand the factors and challenges of the burn population. Some consideration may also be given to the validation of scales over the burn recovery process (e.g., acute management, inpatient hospitalization, outpatient management, etc.), as concerns, coping, adaptation, challenges, needs, and support may all change along this course. There are fewer scales available and validated for pediatric burn survivors. Noted challenges include the limited literacy and numeracy of younger burn survivors. Proxy-reports should be viewed with caution, as caregivers have been found to report a slightly worse severity of symptoms than children across all domains.

## Figures and Tables

**Table 1:** Administration, Psychometric, and Utility Summaries of Mental Health Scales.

Scales	Number of Items	Ages	Administration	α	RCI	Cut Point	LR+	PPV
***Depression***								
BDI-II	21	13–80	Self-report	0.89	3.31/3.94	13	3.91	0.23
HAM-D	Varies	Varies	Self-report	0.79	—	—	—	—
CES-D	20	13+	Self-report	0.85	—	16/20	2.90/3.77	0.18/0.22
PHQ	9	12+	Self-report	0.89	3.69/3.11	10	5.87	0.31
QIDS	16	12+	Self-report or clinician-rated	0.79	—	—	—	—
***Anxiety***								
BAI	21	17–80	Self-report	0.91	4.34/5.15	Varies	—	—
HADS	14	12–65	Self-report	Depends on form	—	11	—	—
GAD-7	7	12+	Self-report	0.92	2.22/2.63	8	5.29	—
***Trauma***								
PCL-5	20	Adults	Self-report	Depends on form	5–10	>31	—	—
CAPS-5	30	7+	Structured Interview	0.88	—	—	—	—
PC-PTSD	5	Adults	Self-report	—	—	3	5.47	—
ITSS	9	Adults	Self-report	—	—	2	—	—
***Suicide***								
ASQ	4	8+	Clinician Administered	—	—	—	—	—
C-SSRS	Varies	5+	Clinician Administered	—	—	—	—	—
BHS	20	17–80	Self-report	0.88	5.63/6.69	9	1.38	—

**Table 2:** Administration Summaries of General Health and Psychosocial Stressor Scales Used with Burn Survivors.

Scales	Number of Items	Ages	Validated for Burn Population	Administration	α
***General Health***					
PROMIS-29	Varies	Adults	Yes	Self-report or proxy rating	0.87–0.97
BSHS-B	40	Adults	Yes	Self-report	0.75–0.93
***Body Image***					
SWAP	14	Adults	Yes	Self-report	0.87
***Community Integration***					
CIQ-13	13	Adults	Yes	Self-report	0.77–0.80
***Pain***					
NRS	1	17–80	No	Self-report	—
VAS	1	8+	No	Self-report	—
FLACC	5	2 months+	No	Observer rating	—
Wong-Baker	1	3+	No	Self-report	—
Oucher (number)	1	8+	No	Self-report	—
Oucher (picture)	1	3+	No	Self-report	—
PAINAD	5	Adults	No	Observer rating	0.30–0.80
***Pruritus***					
4-D	4	Adults	Yes	Self-report	0.82
Itchman	1	6+	Yes	Self-report	—
***Quality of Life***					
SF-36v2^®^	36	Adults	Yes	Self-report	—
SF-12v2^®^	12	Adults	No	Self-report	—
SF-10™	10	5–18	No	Self-report	—
LIBRE Profile-CAT	Varies	Adults	Yes	Self-report	—
LIBRE Profile-SF	30–60	Adults	Yes	Self-report	—
